# PLK4 is a microtubule-associated protein that self-assembles promoting *de novo* MTOC formation

**DOI:** 10.1242/jcs.219501

**Published:** 2018-11-09

**Authors:** Susana Montenegro Gouveia, Sihem Zitouni, Dong Kong, Paulo Duarte, Beatriz Ferreira Gomes, Ana Laura Sousa, Erin M. Tranfield, Anthony Hyman, Jadranka Loncarek, Monica Bettencourt-Dias

**Affiliations:** 1 Cell Cycle Regulation Laboratory, Instituto Gulbenkian de Ciência, Rua da Quinta Grande 6, Oeiras, 2780–156, Portugal; 2 Laboratory of Protein Dynamics and Signalling, National Institutes of Health/National Cancer Institute/Center for Cancer Research, Frederick, MD 21702, USA; 3 Max Planck Institute of Molecular Biology and Genetics, Pfotenhauerstrasse 108, 01307 Dresden, Germany

**Keywords:** PLK4, MTOCs, *In vitro* reconstitution, Microtubule nucleation, PCM, Centrosome, *De novo* assembly, Supramolecular assembly

## Abstract

The centrosome is an important microtubule-organising centre (MTOC) in animal cells. It consists of two barrel-shaped structures, the centrioles, surrounded by the pericentriolar material (PCM), which nucleates microtubules. Centrosomes can form close to an existing structure (canonical duplication) or *de novo*. How centrosomes form *de novo* is not known. The master driver of centrosome biogenesis, PLK4, is critical for the recruitment of several centriole components. Here, we investigate the beginning of centrosome biogenesis, taking advantage of *Xenopus* egg extracts, where PLK4 can induce *de novo* MTOC formation ( Eckerdt et al., 2011; Zitouni et al., 2016). Surprisingly, we observe that *in vitro*, PLK4 can self-assemble into condensates that recruit α- and β-tubulins. In *Xenopus* extracts, PLK4 assemblies additionally recruit STIL, a substrate of PLK4, and the microtubule nucleator γ-tubulin, forming acentriolar MTOCs *de novo*. The assembly of these robust microtubule asters is independent of dynein, similar to what is found for centrosomes. We suggest a new mechanism of action for PLK4, where it forms a self-organising catalytic scaffold that recruits centriole components, PCM factors and α- and β-tubulins, leading to MTOC formation.

This article has an associated First Person interview with the first author of the paper.

## Abstract (precis)

**Summary:** PLK4 binds to microtubules and self-assembles into condensates that recruit tubulin and trigger *de novo* microtubule-organising centre formation *in vitro*.

## INTRODUCTION

Centrosomes are important microtubule-organising centres (MTOCs) in animal cells involved in a variety of processes, including cell motility, division and polarity (Sanchez and Feldman, 2017). They are composed of a core structure, centrioles, surrounded by pericentriolar material (PCM), which nucleates and anchors microtubules (MTs) (Paz and Luders, 2017). PCM proteins also associate with other cellular structures to assemble non-centrosomal MTOCs, a less characterised process (Sanchez and Feldman, 2017).

PLK4, a serine-threonine kinase, triggers procentriole formation close to a pre-existing centriole (the canonical pathway), or induces centriole *de novo* formation when centrioles are absent (*de novo* pathway) (Bettencourt-Dias et al., 2005; Habedanck et al., 2005; Rodrigues-Martins et al., 2007). Recent evidence has shown that both pathways use a common but highly conserved set of proteins, PLK4, STIL and SAS6 (encoded by *SASS6*), that are vital players in centriole assembly (Rodrigues-Martins et al., 2007; Loncarek and Khodjakov, 2009; Loncarek and Bettencourt-Dias, 2018). Upon achieving a critical PLK4 concentration threshold at the centriole or in the cytoplasm, PLK4 becomes active (Loncarek and Bettencourt-Dias, 2018; Lopes et al., 2015) determining the place of biogenesis. Then, PLK4 recruits and phosphorylates STIL, which in turn recruits SAS6 to start forming the cartwheel structure of the centriole (Zitouni et al., 2016). Recently, it was demonstrated that PLK4 also promotes MT nucleation and is essential for spindle assembly in an acentriolar mouse embryo, suggesting that it also contributes to acentriolar MTOC formation (Bury et al., 2017; Coelho et al., 2013).

How PLK4 drives *de novo* MTOC formation is not understood. To study PLK4 function in acentriolar systems, we used both *in vitro* systems and acentriolar *Xenopus* extracts, where PLK4 is sufficient to generate *de novo* MTOCs (Eckerdt et al., 2011; Zitouni et al., 2016). We show that *in vitro* PLK4 self-organises into supramolecular assemblies that recruit tubulin and promote nucleation. In *Xenopus* extracts, PLK4 assemblies recruit STIL, γ-tubulin and α- and β-tubulins (hereafter denoted α/β-tubulin), forming acentrosomal MTOCs *de novo*. Thus, PLK4 plays an important role in forming both centriole-containing and acentriolar MTOCs. It is important to explore the non-canonical functions of PLK4 to better understand the first events of *de novo* MTOC formation.

## RESULTS

### PLK4 self-assembles into condensates that concentrate soluble tubulin *in vitro*

We wanted to understand how PLK4 drives *de novo* MTOC formation. Recently, Woodruff and colleagues used a minimal set of *C. elegans* proteins to reconstitute a functional MTOC *in vitro*. The PCM scaffold protein, SPD-5, self-assembles into spherical assemblies named condensates, which concentrate homologs of XMAP215 and TPX2, resulting in tubulin recruitment and acentrosomal MTOCs formation (Woodruff et al., 2017).

**Fig. 1.**
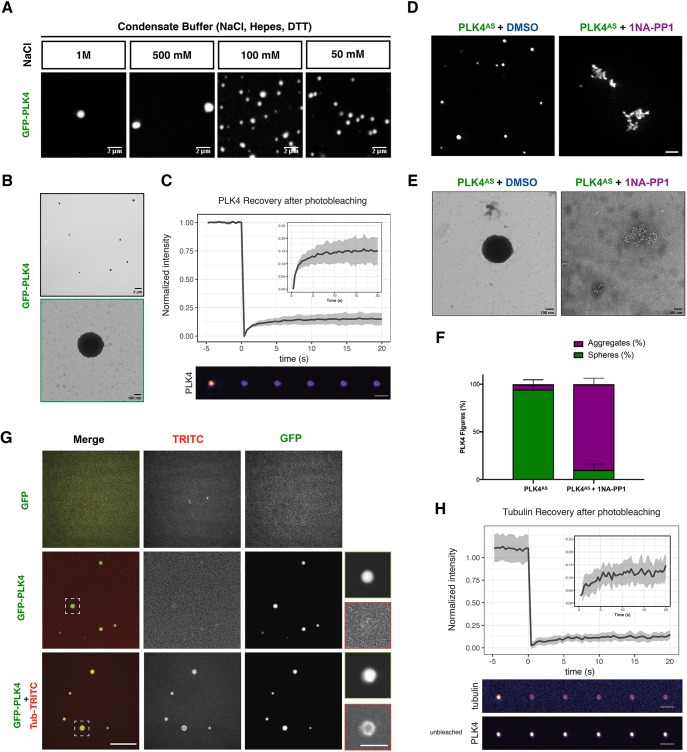
**PLK4 self-assembly is dependent on its kinase activity and PLK4 assemblies concentrate tubulin *in vitro*.** (A) Representative confocal images of GFP–PLK4 assemblies formed at different concentrations of NaCl. Scale bars: 2 μm. (B) Electron microscopy (EM) images showing PLK4 assemblies *in vitro*. Scale bar: 2 μm (top); 100 nm (bottom). (C) Fluorescence intensity recovery after photobleaching (FRAP) (mean±s.d.; %) analysis of PLK4 assemblies *in vitro*. The inset to the graph shows a magnification of the recovery plot; grey shading indicates ±s.d. Scale bar: 2 μm. (D) Confocal images representing GFP–PLK4^AS^ in the absence or presence of 1-Naphthyl-PP1 (1NA-PP1, PP1 analogue). DMSO was used as a control for 1NA-PP1. Scale bar: 5 µm. Note that, in presence of 1NA-PP1, GFP–PLK4 forms disorganised structures. (E) EM images of GFP–PLK4^AS^ in the presence or absence of 1NA-PP1. Scale bars: 100 nm. (F) Quantification of sphere-like assemblies versus aggregates obtained from EM data. Three independent experiments were counted. (G) Confocal images of GFP–PLK4 assembly formation in the absence or presence of Rhodamine-labelled tubulin (500 nM). GFP was used as a control. Scale bars: 5 µm; insets, 2 µm. (H) FRAP analysis as in C of tubulin coating PLK4 spheres *in vitro*, showing that they have little dynamicity. Scale bars: 2 μm.

To explore a minimal system to study PLK4 function, we expressed GFP-tagged *Xenopus* PLK4 in SF9 insect cells using recombinant baculovirus (Fig. S1A). Purified PLK4 is active, as shown by western blotting using an antibody against threonine 170 in its activation loop, within the kinase domain (Fig. S1B) (Lopes et al., 2015). We were surprised to observe that purified active GFP–PLK4 self-assembles *in vitro* into abundant sphere-like structures, when lowering the concentration of NaCl in the buffer (Fig. 1A). The PLK4 sphere-like assemblies were visible as dense bodies under the electron microscope (Fig. 1B; Fig. S1C,D), and recovered very little after photo-bleaching (∼10% recovery; Fig. 1C), suggesting that, similar to what is seen for SPD-5 spherical assemblies (Woodruff et al., 2017), PLK4 assemblies coarsen and become less dynamic with time, with no internal rearrangement. It is possible that PLK4 assemblies form through multimerisation, as different regions of PLK4 can dimerise (Jana et al., 2014). We observed that PLK4 retains its activity while forming these assemblies (Fig. S1D,E). Hereafter, owing their similarity with SPD-5 assemblies, we will call PLK4 assemblies, condensates.

Given that the ability of PLK4 to form centrioles in cells and MTOCs in *Xenopus* extract requires its kinase activity (Rodrigues-Martins et al., 2007; Zitouni et al., 2016), we asked whether this was also the case for PLK4 condensates. We found that recombinant GFP–PLK4^AS^ (L89A/H188Y), which can specifically fit bulky ATP analogues, making it sensitive to ATP analogue inhibitors, for example, 1NA-PP1 (Bishop et al., 2000; Zitouni et al., 2016), also self-assembles (Fig. 1D; Fig. S1F). Importantly, as a control, 1NA-PP1 impairs formation of these structures as observed by confocal and electron microscopy (EM) (Fig. 1D–F), but not the structures generated by wild-type PLK4 (PLK4^WT^), which were only impaired by the PLK4 inhibitor centrinone (Wong et al., 2015) (Fig. S1F). Instead of robust spherical structures, inactivated PLK4 assembles into an amorphous network with no regular shape (Fig. 1D–F), comparable to what is seen with PLK4^WT^ treated with centrinone or with λ-phosphatase for 1 h at room temperature (Fig. S1F–G). Taken together, these data suggest that PLK4 needs to be in its active conformation to self-assemble into sphere-like structures *in vitro* (Fig. 1D–F; Fig. S1).

We next asked whether PLK4 condensates could form an MTOC by incubating it with α/β-tubulin. We observed that PLK4 condensates can recruit and concentrate α/β-tubulin (Fig. 1G). A full tubulin bleach around PLK4 condensates revealed that tubulin does not recover (∼10% recovery; Fig. 1H), suggesting they provide a solid platform for immobile tubulin dimers (Fig. 1H), as previously observed for tau droplets that enhance tubulin assembly (Hernández-Vega et al., 2017).

### PLK4 binds MTs *in vitro*

**Fig. 2.**
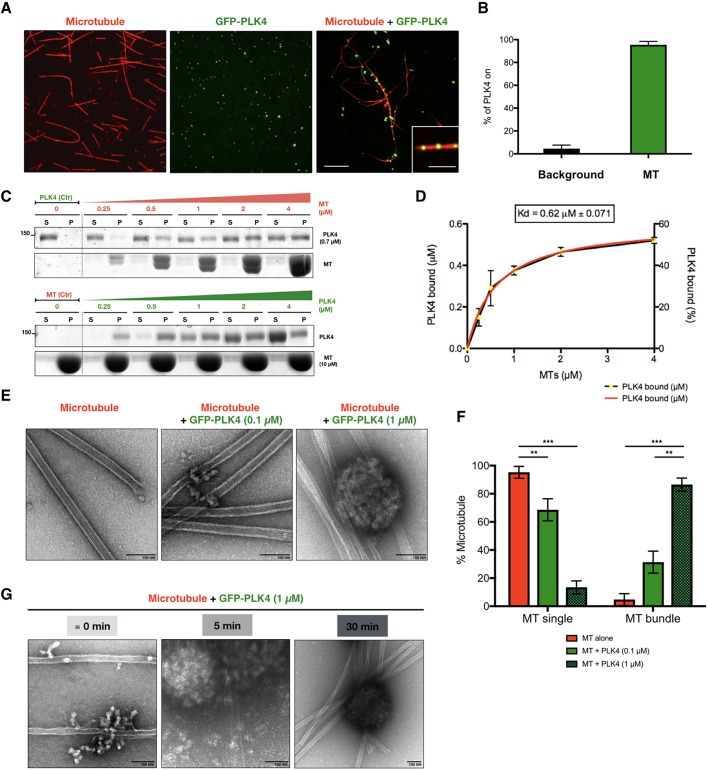
**PLK4 is a microtubule-associated protein that promotes microtubule bundling *in vitro*.** (A) Confocal images of Taxol-stabilised MTs alone (Rhodamine-labelled tubulin, red), recombinant purified GFP–PLK4 alone (green) and the mixture of both, showing association of PLK4 condensates to MTs. Scale bar: 5 μm; inset, 2 µm. (B) Quantification of PLK4 assemblies (mean±s.d.; %) associated to MTs compared to free PLK4 in the background (*N*=3, *n*=100 spot/condition). (C) MT-pelleting assays. The two assays are showing either a constant PLK4 concentration (0.7 μM) mixed and incubated with different MT concentrations (0 to 4 μM) or increasing amounts of GFP–PLK4 (0 to 4 µM) in the presence of a constant MT concentration (10 µM). The Coomassie Blue-stained gel is showing supernatant (S) and pellet (P) for each condition. (D) Quantitative analysis of binding properties between PLK4 and MTs. Note that the dissociation constant (*K*_d_) for PLK4, determined by best fit to the data (red curve), is 0.62±0.071 μM. Note that the dotted line is the real data (mean±s.e.m.) and the red line is the fitted curve to derive constants. The data were collected from three independent experiments. (E) EM images showing MTs alone or MTs incubated with two concentrations of PLK4 (0.1 µM and 1 µM). Scale bars: 100 nm. (F) Percentage of single or bundled MTs quantified from EM data in presence of PLK4 (0.1 µM or 1 µM); MTs alone are used as a control. Results are mean±s.e.m. scored using 30 images per condition obtained from three independent experiments each (***P<0.001; **P<0.01, Student's *t*-test). (G) Time course of PLK4 (1 µM) incubated with MTs. Note that PLK4 binds to MTs before PLK4 condensates are formed (“≡” means ∼0 min, as feasible experimentally). Scale bars: 100 nm.

Given our intriguing observation that PLK4 condensates can recruit α/β-tubulin, we inquired whether PLK4 could bind polymerised α/β-tubulin and promote MT stabilisation, which could contribute to its ability to promote MTOC formation. We observed that GFP–PLK4 condensates associate with stable MTs *in vitro* (Fig. 2A). The great majority of PLK4 condensates *in vitro* are associated with MTs (∼95.4%) (Fig. 2A,B). We then asked whether PLK4 binds directly to MTs. Polymerised MTs pelleted (P) with bound PLK4 whereas the unbound fraction remained suspended in solution (S). We observed that purified PLK4 co-pelleted with the MT fraction *in vitro*. To calculate the binding dissociation constant (*K*_d_), we performed pelleting assays with a constant PLK4 concentration (0.7 μM) and increasing MT concentrations (0 to 4 μM) (Fig. 2C). Reciprocally, we performed the same assay using a constant amount of MTs (10 μM) and increasing amounts of PLK4 (0 to 4 μM) until the saturation point was reached (Fig. 2C). We plotted PLK4 bound to MTs versus MT concentration, from three independent experiments. The calculated *K*_d_ is the concentration of MTs that is required to sediment half of PLK4 (Fig. 2D). These data strongly indicate that PLK4 is a MT-associated protein that binds MTs directly with high affinity (*K*_d_=0.62±0.071 μM, mean±s.e.m.). The presence of PLK4 led to an increase of MT bundles (Fig. 2E,F), as many other MT-binding proteins do (Walczak and Shaw, 2010). As MT bundles stabilise MT dynamics, perhaps PLK4 promotes MT stabilisation and nucleation (Brandt and Lee, 1994; Umeyama et al., 1993).

Importantly, PLK4 kinase activity was not required for both α/β-tubulin recruitment (Fig. S2A) and MT binding (Fig. S2B,C), suggesting that they rely on a scaffold but not on the catalytic function of PLK4. A time course using PLK4 and MTs showed that PLK4 binds to MTs independently of the PLK4 sphere-like condensate formation (Fig. 2G). These results show a new role for PLK4 as a MT-associated protein involved in MT stabilisation and possibly in MT nucleation.­

### PLK4 condensates function as MTOCs in *Xenopus* extracts

**Fig. 3.**
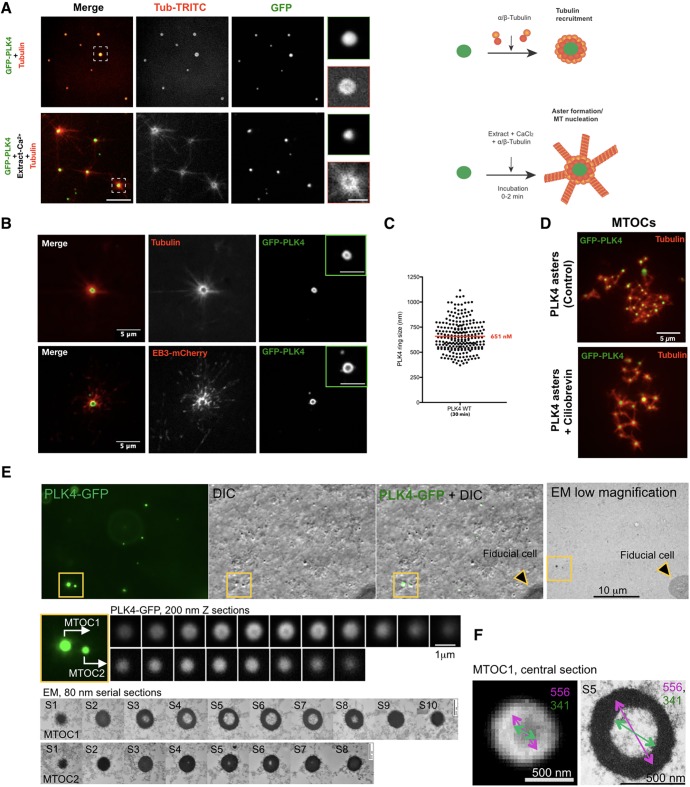
**PLK4 condensates form *de novo* MTOCs in *Xenopus* extracts independently of motor proteins and mimic centrosomes *in vivo*.** (A) Top, Condensates are formed by mixing GFP–PLK4 with Rhodamine-labelled tubulin in the condensate buffer (*in vitro*). Bottom, Ca^2+^-released MII egg extract-containing Rhodamine-labelled tubulin was added to these assemblies. Note that nucleation was observed instantly after the addition of the mixture (0–2 min). Scale bar: 5 µm, insets, 2 µm. (B) Confocal images showing MTOC formation in *Xenopus* MII Ca^2+^-released extracts in the presence of recombinant GFP–PLK4 (green). MTs are visualised by means of Rhodamine-labelled tubulin (upper panel) and EB3–mCherry (lower panel). MT plus-ends visualised by means of EB3–mCherry point out the edge of the aster. Insets show PLK4 as a ring-like structure (Movie 1). Scale bars: 5 μm, insets 2 μm. (C) Quantification of the size (nm) of GFP–PLK4 ring-like structure after 30 min of incubation. GFP–PLK4 rings were measured from three independent experiments. (D) PLK4 aster formation is independent of dynein. Confocal images of PLK4 asters are shown in the control and in the presence of ciliobrevin (a dynein inhibitor). Scale bar: 5 μm. (E) Correlative light electron microscopy analysis of PLK4 MTOCs. PLK4–GFP signals were first visualised by fluorescence and DIC microscopy, and then by EM. A series of 200 nm sections (confocal) and 80 nm EM sections are presented for two MTOCs (yellow box, MTOC1 and MTOC2). Scale bars: 10 μm, 1 μm and 500 nm. (F) Measurements of the central sections of MTOC1 (section S5 in E). Scale bars: 500 nm.

We asked whether PLK4 condensates could promote MT nucleation in the presence of a more complex environment. It was previously shown that PLK4 induces *de novo* MTOC formation after M-phase exit in *Xenopus* extracts (Eckerdt et al., 2011; Zitouni et al., 2016). We observed that PLK4 condensates, initially formed *in vitro*, can nucleate MTs when *Xenopus* extracts were added, suggesting that they can act as a scaffold forming an active MTOC, given the right factors supplied (Fig. 3A).

Next, we investigated whether GFP–PLK4 would also form assemblies in the more physiological context of *Xenopus* extracts. We used Rhodamine–tubulin and EB3–mCherry (EB3 is also known as MAPRE3) to visualise GFP–PLK4-induced MT nucleation (Fig. 3B; Movie 1). PLK4 assemblies were also formed in this context, bearing variable sizes (Fig. 3C), which are akin to centrosome size (300 to 1000 nm; average size ∼650 nm). These MTOCs contained GFP–PLK4 at their core, surrounded by tubulin (Fig. 3B), which is similar to what we observed *in vitro* (Fig. 1G), suggesting that the same type of assemblies are present *in vitro* and in extracts.

We next investigated whether the PLK4-mediated formation of MTOCs is dependent on dynein. Purified centrioles and DMSO asters were used as controls. While DMSO asters are destroyed in the presence of dynein inhibitors, such as vanadate and ciliobrevin, centrioles, as expected, remained capable of nucleating MTs (Fig. S3A). In the case of PLK4-driven MTOCs, asters formed in a dynein-independent manner (Fig. 3D; Fig. S3A), suggesting they are indeed self-assembled, and more similar to centrosomes.

Importantly, even in the extract, we observed that PLK4 had an affinity for tubulin. We used interphase extracts to obtain stable asters from GFP-containing centrioles, and supplemented with soluble PLK4. We observed some PLK4 associating with MTs (Fig. S3B; Movie 2). However, the majority of PLK4 was localised at the centre of the MTOCs, as seen *in vitro*, suggesting a core role for PLK4 in MT organisation.

We next examined whether PLK4 condensates could form centrioles in *Xenopus* extracts by performing correlative light electron microscopy (CLEM) (Fig. 3E). Unexpectedly, we observed no centrioles. Instead, PLK4–GFP assemblies corresponded to hollow or sometimes filled spheres of variable sizes (100–600 nm; Fig. 3E,F). These structures agreed with the appearance of PLK4 MTOCs as observed by confocal microscopy. We speculate that the formation of PLK4 assemblies that appear hollow in the *Xenopus* extracts, which were not observed *in vitro*, could be due to the presence of additional factors present in this system. Notwithstanding this difference, we conclude that PLK4 MTOCs nucleate MTs, analogous to bona fide centrosomes.

### PLK4 MTOCs recruit STIL and γ-tubulin in *Xenopus* extracts, leading to MT nucleation

**Fig. 4.**
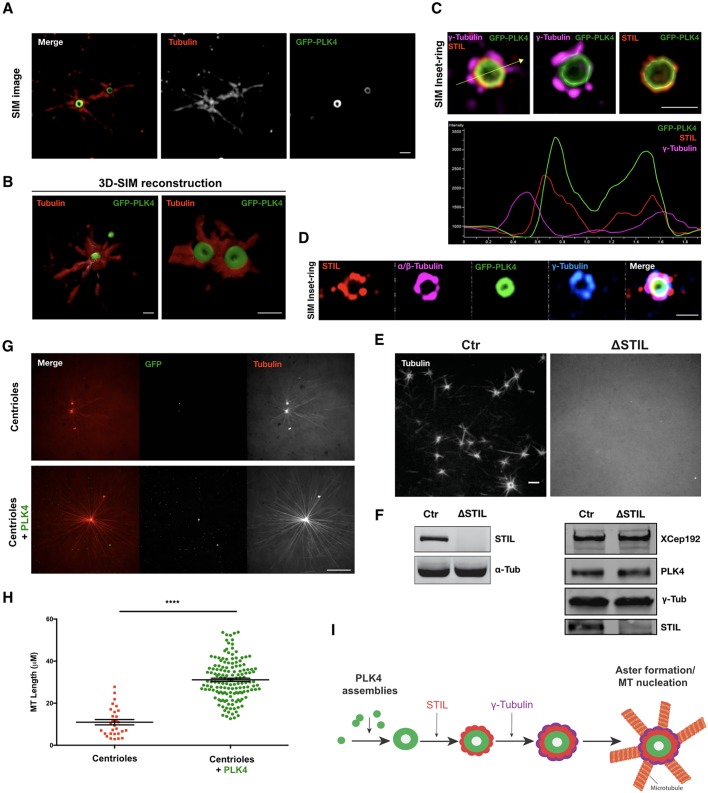
**PLK4 MTOCs can recruit STIL and γ-tubulin in Ca^2+^-released *Xenopus* extracts and are able to enhance centrosomal MT nucleation.** (A) 3D-SIM images showing a ring-like structure of PLK4 MTOCs formed in Ca^2+^-released *Xenopus* extracts. α-tubulin and GFP–PLK4 are presented in red and green, respectively. Scale bar: 1 µm. (B) 3D reconstitution of PLK4 asters (Movie 3). Scale bars: 1 µm. (C) SIM images and profile across the arrow showing the colocalisation of STIL (red), γ-tubulin (magenta) and GFP–PLK4 (green) within PLK4 MTOCs. Scale bar: 1 µm. (Movie 4). (D) SIM images showing that GFP–PLK4 (green), STIL (red), α/β-tubulin (magenta) and γ-tubulin (blue) colocalise with PLK4 MTOCs. Scale bar: 1 µm. (E) Confocal images showing PLK4-induced MTOCs containing Rhodamine–tubulin in control extracts (Ctr) and STIL-depleted extract (ΔSTIL). Scale bar: 1 µm. (F) Western blots showing depletion of STIL in the extracts used in E. The total level of proteins in these extracts is shown using antibodies against XCep 192, γ-tubulin and PLK4. (G) PLK4 enhances MT nucleation. Confocal images showing MT nucleation using purified centrioles labelled with GFP–centrin incubated in *Xenopus* interphasic extract in the presence or absence of GFP–PLK4 (Rhodamine-labelled tubulin, red; centriole and PLK4, green). Images were taken after 30 min incubation (Movies 5 and 6). Scale bar: 5 µm. (H) Quantifications of MTs length (μm) visualised from the centrioles (GFP–centrin MTOCs) in the presence or absence of GFP–PLK4. MTs were measured from two independent experiments, where four different MTOCs were analysed (the total number of MTs measured in the presence of GFP–PLK4 was 225). The statistical data are presented as mean±s.d. ****P<0.0001, (Mann–Whitney U-test). (I) Representative schematic of PLK4 MTOC formation in *Xenopus* extracts.

To further understand the capacity of PLK4 condensates to form MTOCs, we asked whether their function depends on their ability to recruit other PLK4 partners and/or modulators of α/β-tubulin function. First, we used structured illumination microscopy (SIM) to characterise PLK4 MTOCs in *Xenopus* extracts. We could observe a GFP–PLK4 ring structure in the crosscuts (Fig. 4A) and sphere structure after 3D rendering (Fig. 4B; Movie 3).

STIL is the best-characterised substrate of PLK4 and formation of a PLK4–STIL complex triggers centriole biogenesis (Arquint et al., 2015; Kratz et al., 2015; Moyer et al., 2015; Ohta et al., 2014). γ-tubulin is a highly conserved protein, which is associated to most characterised MTOCs (O'Toole et al., 2012; Teixido-Travesa et al., 2012). Most γ-tubulin in animal cells appears to exist as γ-tubulin ring complexes (γ-TuRCs) and to be important for centrosome-dependent MT nucleation (Wiese and Zheng, 1999). Interestingly, GCP6 (also known as TUBGCP6), a γ-TuRC member, is also a PLK4 substrate (Bahtz et al., 2012; Martin et al., 2014). Thus, we investigated whether STIL and γ-tubulin associate with PLK4 MTOCs by means of immunofluorescence. We found STIL and γ-tubulin to associate with PLK4 MTOCs in a spatially ordered manner (Fig. 4C,D) so that PLK4 formed an inner layer, followed by STIL and γ-tubulin (Fig. 4C,D; Fig. S4A, Movie 4). Depletion of STIL prevented the formation of PLK4-induced MTOCs in extracts, suggesting that the same pathway triggers the formation of centrioles and acentriolar MTOCs (Fig. 4E,F) (Zitouni et al., 2016). Owing to the lack of specific antibodies against *Xenopus* proteins, we were unable to analyse whether other centrosomal proteins associate with PLK4 MTOCs.

### PLK4 leads to the formation of stronger centrosomal MTOCs

PLK4 might also promote MT nucleation in different ways in centrosomal MTOCs. It was recently proposed that MT stabilisers can promote MT nucleation in cells (Roostalu et al., 2015; Roostalu and Surrey, 2017; Wieczorek et al., 2015). The authors proposed that MT stabilisers control the nucleation efficiency by stabilising the MT centre or ‘nucleus’, either by providing a template for assembly or by promoting longitudinal or lateral tubulin–tubulin interactions. Additionally, by locally recruiting α/β-tubulin, PLK4 assemblies could promote high α/β-tubulin concentrations around the MTOC centre, which is known to lead to enhanced MT assembly, as it is observed in BuGZ and SPD-5 condensates (Jiang et al., 2015; Woodruff, 2018; Woodruff et al., 2017).

To address this hypothesis, we used purified GFP–centrin centrioles from HeLa cells and incubated them with *Xenopus* extracts. Shortly after addition to interphasic *Xenopus* extract, purified centrioles could recruit PCM components and nucleate MTs. However, when we added GFP–PLK4, we observed a very significant increase in MT nucleation capacity, MT elongation and a decrease in MT dynamics, suggesting their stabilisation (Fig. 4G,H; Movie 5 and 6). We observed the same effect in *Xenopus* extracts upon M-phase release (Fig. S4B,C).

## DISCUSSION

The centrosome is an important MTOC in animal cells. Centrosomes can form close to an existing structure (canonical duplication) or *de novo* (Bettencourt-Dias et al., 2005; Habedanck et al., 2005; Rodrigues-Martins et al., 2007). However, the mechanisms underlying *de novo* centrosome formation remain unclear.

In this study, we show that PLK4 self-organises into condensates that can recruit α/β-tubulin. However, this did not lead to MTOC formation, suggesting that other molecules are needed for the observed ability of PLK4 to form centrioles and MTOCs in extracts and *in vivo* (Arquint et al., 2015; Baumbach et al., 2015; van Breugel et al., 2011).When PLK4 is added to *Xenopus* extracts it forms condensates, which can further recruit STIL and γ-tubulin, and possibly other components, forming a layered MTOC, similar to centriole-containing centrosomes*.* Since GCP6 (a substrate of PLK4) is required to recruit the γ-TuRC to centrosomes, perhaps it can recruit γ-TuRC to PLK4 assemblies (Bahtz et al., 2012; Oriolo et al., 2007; Teixido-Travesa et al., 2012). Additionally, CPAP, a binding partner of STIL involved in MT stabilisation, can indirectly enhance MT nucleation from PLK4 MTOCs (Sharma et al., 2016). The ability to mimic the layered centrosome *in vitro* opens new ways of understanding PCM assembly.

In addition, we observed that PLK4 binds to MTs and induces bundling formation. PLK4 kinase activity is not needed for its binding to MTs and recruitment of α/β-tubulin but is needed for PLK4 self-assembly into sphere-like structures and for forming an MTOC in *Xenopus* extracts. These observations suggest that PLK4 may have both scaffold and catalytic functions in MTOC formation. Further work is needed to understand those two functions and how they are coordinated. Taken together, these results suggest a new role for PLK4, in which it binds MTs and serves as a scaffold to concentrate α/β-tubulin, increasing the local tubulin critical concentration, which in turn could promote MT nucleation. Similar conclusions were drawn by a recent study showing similar structures into which tubulin can accumulate, enhancing MT polymerisation (Jiang et al., 2015; Woodruff, 2018; Woodruff et al., 2017).

PLK4 forms condensates that recruit several important components in MT nucleation, including γ- and α/β-tubulin. We suggest that this lowers the critical concentration of spontaneous MT nucleation leading to MTOC formation (Fig. 4I). Our data strongly suggest that, in extract, PLK4-driven MTOCs are initiated from PLK4 condensates, and in contrast to acentrosomal MTOCS, do not depend on motor-based self-assembly of MT minus-end-bound material (acentrosomal MTOCs) (Compton, 1998; Mitchison, 1992; Sanchez and Feldman, 2017), where motor proteins, for example, dynein, play a crucial role in forming acentrosomal MTOCs (Gaglio et al., 1997, 1996).

Furthermore, PLK4 MTOCs exhibit a layered organisation, as it was shown to exist in the interphasic centrosome in animal cells (Lawo et al., 2012; Mennella et al., 2012; Sonnen et al., 2012). It is thus possible that, even upon the presence of centrioles, PLK4 could promote the formation of assemblies that concentrate components critical to form centrioles: including STIL, γ-tubulin and α/β-tubulin. PLK4 itself, given that it binds and stabilises MTs, can also promote further MT nucleation (Roostalu and Surrey, 2017), hence contributing to daughter centriole assembly. Indeed, we observed that PLK4 enhances centrosomal MTOC nucleation. These observations are very similar to those from Popov and colleagues with XMAP215, a processed MT polymerase that plays an important role in MT nucleation, in addition to γ-tubulin (Brouhard et al., 2008; Popov et al., 2002). Unlike XMAP215, and like Aurora B (Tsai and Zheng, 2005), PLK4-coated beads do not nucleate MTs in the presence of tubulin (data not shown) below a critical concentration (<20 µM) (Wieczorek et al., 2015). One explanation could be that PLK4-coated beads alter PLK4 conformation, preventing the formation of PLK4 assemblies and blocking MTOC formation. In addition, Aurora B kinase activity is essential for MTOC formation, identical to what happens with PLK4 (Tsai and Zheng, 2005). Altogether, our observations suggest a mechanism by which PLK4 promotes MT nucleation and stabilisation in centrosomal and acentrosomal systems. Further work is needed to understand how PLK4 condensates are formed, and whether they are formed at the site of centriole birth on mother centrioles.

In conclusion, our work shows that PLK4 assembles into condensates, which recruit downstream centrosomal components that are important for both centriolar duplication and microtubule nucleation. These condensates form only in the presence of active PLK4. However, the recruitment of downstream components is independent of the kinase activity. This suggests that PLK4 has both catalytic and scaffold roles in MTOC formation. Although further work is needed to clarify these mechanisms, this study provides new ways to understand PCM assembly and *de novo* centrosome formation.

## MATERIALS AND METHODS

### PLK4 protein purification

The full-length *Xenopus* PLK4 gene lacking a stop codon was amplified by PCR and inserted into in-house-designed baculoviral expression plasmids (pOCC series) to generate the following construct: MBP-PreScission::PLK4::-mEGFP::PreScission-6×His. The protein was expressed in SF9 insect cells in ESF921 (Expression Systems, LLC #94-001F) or SF21 insect cells in Sf-900 (Gibco, #12682019) and harvested 72 h post infection. Cells were collected, washed and resuspended in harvest buffer (50 mM Tris-HCl pH 7.4, 150 mM NaCl, 30 mM imidazole, 1% glycerol) plus protease inhibitors (1 mM PMSF, 100 mM AEBSF, 0.08 mM Aprotinin, 5 mM Bestatin, 1.5 mM E-64, 2 mM Leupeptin, 1 mM Pepstatin A) (Calbiochem) and frozen in liquid nitrogen. The protein was purified using a two-step purification protocol described in Woodruff and Hyman (2015). PLK4 clarified lysate was incubated first with Ni-NTA agarose beads followed by a second incubation with amylose resin. The MBP and 6×His tags were cleaved and PLK4 was eluted by overnight incubation with PreScission protease. PLK4 was concentrated with a 50K Amicon Ultra centrifugal concentrator (Millipore), aliquoted and flash frozen in liquid nitrogen. The lysis and final elution buffer used (called ‘PLK4 buffer’) contained 50 mM Tris-HCl pH 7.4, 500 mM NaCl, 0.5 mM DTT, 1% glycerol and 0.1% CHAPS. The intermediate elution buffer from the Ni-NTA beads contained an additional 250 mM imidazole (Woodruff and Hyman, 2015). As a control, we used the construct 6×His::Z-tag::TEV::SBP::mEGFP (created in our laboratory) to express GFP, which is purified as previously mentioned.

### *In vitro* PLK4 self-assembly

PLK4 assemblies were formed by adding purified GFP–PLK4 (1 µM) to the condensate buffer (150 mM NaCl, 25 mM HEPES pH 7.4 and 1 mM DTT). PLK4 was incubated for 5 to 10 min at room temperature (or at different temperatures when checking their effect on PLK4 assemblies; Fig. S2C) and then imaged using a spinning disc CSU-X1 (Yokogawa) confocal scan head coupled to a Nikon Eclipse Ti-E and controlled using MetaMorph 7.5 (Molecular Devices). For tubulin recruitment, Rhodamine–TRITC-labelled tubulin (Cytoskeleton; 500 nM) was added to the condensate buffer. We used BRB80 buffer (see below) as a control. For the experiments using inactive PLK4, 1NA-PP1 (Cayman Chemical, CAYM10954-1, 100 µM) was used to inactivate PLK4^AS^; DMSO was used as a control. Dephosphorylation of PLK4 was performed using the typical reaction protocol for λ-phosphatase, incubated for 1 h at room temperature and processed either for confocal imaging or for electron microscopy. A sample was taken to check for PLK4 activity by western blotting using a rabbit antibody against phosphorylated threonine-170 raised in our laboratory (see below).

### Microtubule pelleting assay

Tubulin was polymerised into MTs stabilised with 20 µM Taxol in BRB80 buffer [25 mM HEPES, pH 6.8, 2 mM MgCl_2_, 1 mM EGTA and 0.02% (v/v) Tween 20], and quantified by absorbance measurements at 280 nm. Various concentrations of MTs were mixed with constant concentrations of PLK4 in BRB80 buffer or vice versa. Samples (final volume of 40 µl) were equilibrated at 37°C for 25 min, centrifuged in an Airfuge at 86,000 ***g*** for 30 min, and both the supernatant (S) and pellet (P) collected and resuspended in SDS sample buffer, and equal amounts of supernatant and pellet were run on 4–20% Tris-HCl gradient gels (Bio-Rad). Gels were stained with Coomassie Blue or western blotted to detect MTs and PLK4. Quantification of the relative amounts of PLK4 in supernatants and pellets was performed using ImageJ (National Institutes of Health, Bethesda, MD). The dissociation constants measured by MT co-sedimentation represent the average and propagated error from three independent experiments.

### *In vitro* PLK4 and microtubule bundling on confocal assay

Taxol-stabilised MTs seeds were incubated with GFP–PLK4 (1 µM) for 15 min at 37°C and then mounted in a slide and observed on a confocal microscope. Taxol MT seeds were performed as previously described (Honnappa et al., 2009). Briefly, lyophilised 1 mg of tubulin (Cytoskeleton) was resuspended in 100 µl of BRB80 buffer, 1 mM GTP and 4 mM MgCl_2_ and incubated for 30 min at 37°C. After 20 min, Taxol was added to a final concentration of 20 µM. Finally, Taxol-MTs were stored at room temperature.

### Fluorescence recovery after photo bleaching

Tubulin recruitment by PLK4 assemblies was performed as described previously (Woodruff et al., 2017). For the FRAP analysis of the *in vitro* PLK4 assemblies (alone or coated with tubulin), samples were mounted in an imaging chamber after 10 min of incubation at room temperature. FRAP experiments were performed on the same spinning disc confocal microscope mentioned above using a 100×1.49 NA oil immersion objective. Laser micro-irradiation was performed with a 491 nm or 561 nm laser with a 100 ms exposure time. Tubulin coating PLK4 was bleached for 15 ms and images were taken at 0.4 s intervals. Analysis of the recovery curves and the half-time recovery were carried out with the FIJI/ImageJ macro (http://imagej.net/Analyze_FRAP_movies_with_a_Python_script). The normalised data were parsed and plotted using R software.

### Electron microscopy negative staining assay

For the electron microscopy assays, GFP–PLK4^WT^ or GFP–PLK4^AS^ was used to visualise the structures when the proteins were incubated in different buffers and at different conditions (PLK4 buffer BRB80 with or without DMSO, in the presence or absence of microtubules, and incubated at different temperatures). A time course (0 to 10 min) was also performed to observe the evolution of the assemblies. For acute inactivation of PLK4, 1NA-PP1 or centrinone (PLK4 inhibitor) was added simultaneously with GFP–PLK4^AS^ or GFP–PLK4^WT^, respectively. When PLK4 was mixed with microtubules, the incubation was performed at 37°C for 30 min. After the reaction, samples were adhered to glow discharged copper 150 mesh grids coated with 1% (w/v) formvar (Agar Scientific^®^) in chloroform (^®^VWR) and carbon. Following attachment, samples were rinsed with distilled water and stained with 2% (w/v) uranyl acetate. Electron microscopy images were acquired on a Hitachi H-7650 operating at 100 keV equipped with a XR41M mid-mount AMT digital camera.

### Preparation of *Xenopus laevis* egg extracts and MTOC formation assay

*Xenopus* eggs arrested at metaphase of meiosis II by the activity of the cytostatic factor (CSF) [called (MII)-arrested extracts or CSF extracts] and interphase egg extracts were prepared as previously described (Lorca et al., 2010; Zitouni et al., 2016). Purified PLK4 (0.675 nM) was added to 20 µl of CSF extracts, which were then released into interphase through addition of Ca^2+^ (20 mM). MTOCs were analysed by visualising Rhodamine-labelled porcine tubulin (Cytoskeleton). Depletion of STIL in *Xenopus* extracts was performed as described previously (Zitouni et al., 2016). This research project was ethically reviewed and approved by the Ethics Committee of the Instituto Gulbenkian de Ciência (license reference A007.2010), and by the Portuguese National Entity that regulates the use of laboratory animals [Direção Geral de Alimentação e Veterinária (DGAV); license reference 0421/000/000/2017]. All experiments conducted on animals followed the Portuguese (Decreto-Lei 113/2013) and European (Directive 2010/63/EU) legislations concerning housing, husbandry and animal welfare.

For assays using GFP–centrin-labelled centrosomes, centrosomes were added to interphasic extract or to MII-arrested extract containing Rhodamine-labelled tubulin in the presence or the absence of GFP–PLK4 (1 µM). These extracts were incubated for 30 min (interphasic extract; Fig. 4H) or 15 min (MII released extract; Fig. S4B) at 18°C and visualised by performing confocal microscopy. When GFP–centrin centrosomes were added in the extract in the presence or absence of PLK4, we measured the length of MTs (Fig. 4I) or the intensity of MT nucleation via Rhodamine–tubulin intensity (Fig. S4B). Prism (version 5.0c; GraphPad) was used for statistical analysis and plotting when needed. To visualise PLK4 binding to MTs, as previously done, centrioles labelled with GFP–centrin were added to interphasic extracts and supplemented with further soluble GFP–PLK4 (1 µM).

### Antibodies

Antibodies against STIL (Human SIL antibody, A302-441A, rabbit anti-SIL Antibody, Affinity Purified; dilution 1:1000) and γ-tubulin (GTU-88, T5326 Sigma; dilution 1:500) were purchased from Bethyl Laboratory and Sigma, respectively. Anti-xCEP192 (dilution 1:500) polyclonal antibodies were generated by immunising rabbits with the purified fragments of *Xenopus* Cep192 [amino acids 521–1000 (αCep192-N)]. Antibodies were affinity purified by using the relevant antigens cross-linked to AminoLink Coupling Resin (Thermo Scientific) according to the manufacturer's instructions). Anti-xCEP192 was a gift from Vladimir Joukov (Dana-Farber Cancer Institute, Boston) and XPLK4 was used as in Zitouni et al. (2016). Rabbit anti-phospho-Thr170 antibody (dilution 1:500) was produced by immunising rabbits with a synthetic peptide against the activation T-loop [(K)HYpTLCGTPNY] of the human sequence. Antibodies against histidine (dilution 1:1000; Novagen material number: 70796-3, kit batch number: D00106784) and GFP (dilution 1:1000; Roche Applied Science; catalogue no.: 11814460001, clone name 7.1, 13.1) were also used.

### Super-resolution of PLK4 assemblies in *Xenopus* extracts

Structured illumination microscopy (SIM) of PLK4–GFP assemblies was performed on an N-SIM system, Nikon Inc., equipped with Apo TIRF 100× NA 1.49 Plan Apo oil objective, back-illuminated EMCCD camera (Andor, DU897), and 405, 488, 561 and 640 nm excitation lasers. 100-nm *z* sections were acquired in 3D SIM mode generating 15 images per plan, and reconstructed. *xyz* corrections were applied using the signals of 100 nm multi-spectral fluorescent spheres (Invitrogen) included in the sample.

### Correlative light and electron microscopy

To correlate light and electron microscopy images of PLK4–GFP assemblies, an *in vitro* PLK4–GFP self-assembly reaction mixture was overlaid directly to the coverslips mounted in Attofluor Cell Chambers (Invitrogen; A7816) and kept at 37°C. The coverslips contained previously sparsely seeded fixed HeLa cells. HeLa cells served as landmarks for subsequent identification of target PLK4–GFP assemblies during trimming, sectioning and imaging on for the electron microscope. PLK4–GFP MTOCs were fixed in 2.5% glutaraldehyde, and immediately imaged on an inverted microscope (Eclipse Ti; Nikon, Tokyo, Japan) equipped with a spinning-disc confocal (CSUX Spinning Disk; Yokogawa Electric Corporation, Tokyo, Japan), back-illuminated 13 µm pixel EMCCD camera (Andor, DU888), 100× NA 1.42 Plan Apo objective lens with 1.5× magnifying tube lens, and a 2× lens in front of the confocal head. The positions of GFP–PLK4 assemblies were recorded by acquiring a stack of 200-nm-thick *z* sections in fluorescence mode and then in DIC mode (using Nikon DS-U3 camera). The position of the target PLK4–GFP assemblies and fiducial cells on the coverslip was marked with a diamond scribe, as described previously (Kong and Loncarek, 2015). After fixation, the samples were washed in PBS for 30 min, pre-stained with osmium tetroxide and uranyl acetate, dehydrated in ethanol, and then embedded in Embed 812 resin. 80-nm-thick serial sections were sectioned, transferred onto the formvar-coated copper slot grids, stained with uranyl acetate and lead citrate, and imaged using a transmission electron microscope (H-7650; Hitachi, Tokyo, Japan) operating at 80 kV. For the alignment of serial sections and for image analysis we used Photoshop (Adobe) and Fiji (NIH) software.

## Supplementary Material

Supplementary information
