# Supplementary material for: PLK4 is a microtubule-associated protein that self-assembles promoting de novo MTOC formation
Source: J Cell Sci. 2018 Nov 9;132(4):jcs219501. doi: 10.1242/jcs.219501 (PMC6398482; doi:10.1242/jcs.219501)
Supplement: Supplementary information [file joces-132-219501-s1.pdf]

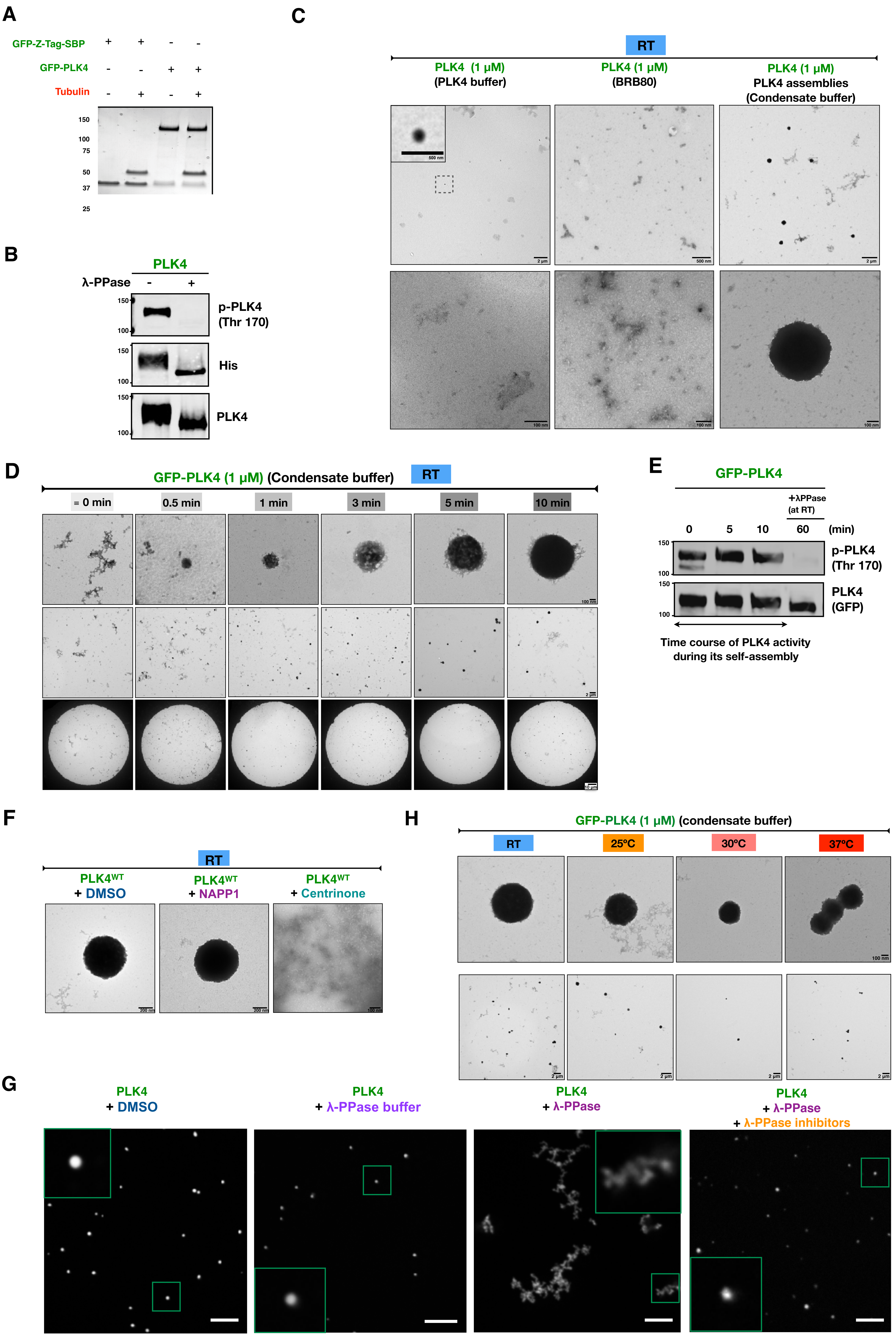

Journal of Cell Science • Supplementary information

Fig. S1. (Related to Fig. 1). Formation of PLK4 supramolecular assemblies is dependent on its activity.

(A) Coomassie-stained gels showing the proteins used in different conditions in Figure 1. (B) Western blots showing PLK4's activity, when treated or not with lambda-phosphatase (λ-PPase), using an antibody against threonine-170 localised within the kinase domain. (C) EM images of PLK4 assemblies in control buffer (*PLK4 buffer* (50 mM Tris-HCl, pH 7.4, 500 mM NaCl, 0.5 mM DTT, 1% glycerol, 0.1% CHAPS) and *BRB80/ MTs buffer* (25 mM HEPES, pH 6.8, 2 mM $MgCl_2$, 1 mM EGTA, 0.02% Tween 20 (v/v)) and in the *Condensate buffer* (150 mM NaCl, 25 mM Hepes (pH 7.4); 1 mM DTT). Note that we detected few small PLK4 assemblies in PLK4 buffer similarly to what is shown in Figure 1A at 1 M and 500 mM of NaCl. (D) Time course of PLK4 assembly formation. Note that PLK4 assemblies grow and become round and dense while forming, with heterogeneous sizes (500 nm to 1 μm). (E) WB showing the activity of PLK4 while forming the assemblies (Thr170 antibody and GFP for total PLK4 protein). Note that we used the λ-PPase treatment for 1 hour at room temperature (RT) to show the efficiency of the dephosphorylation compared to the typical reaction protocol of the phosphatase (30 min at 30ºC; Fig. S1B). (F) EM images showing PLK4-WT assemblies in the control condition (DMSO) and when treated with NAPP1 (specifically inhibiting PLK4-AS) and centrinone (specific inhibitor of PLK4). Note that NAPP1 and centrinone were added simultaneously with the protein for acute inactivation. (G) Confocal images of GFP-PLK4 treated with λ-PPase used according to the typical reaction protocol, incubated for 1 hr at RT. The corresponding controls were performed to check effects of the buffers on PLK4 assemblies. To inactivate λ-PPase we used: 10 mM sodium orthovanadate, 50 mM EDTA, protease inhibitor cocktail 10%, leupeptin and aprotinin 10 μg/ml, added to the buffer simultaneously with the phosphatase for acute inactivation. Scale bars: 5 μm. (H) EM images of PLK4 assembly formation at different temperatures. Note that the structures are stable at 37ºC, the temperature used for the assays with MTs.

Journal of Cell Science • Supplementary information

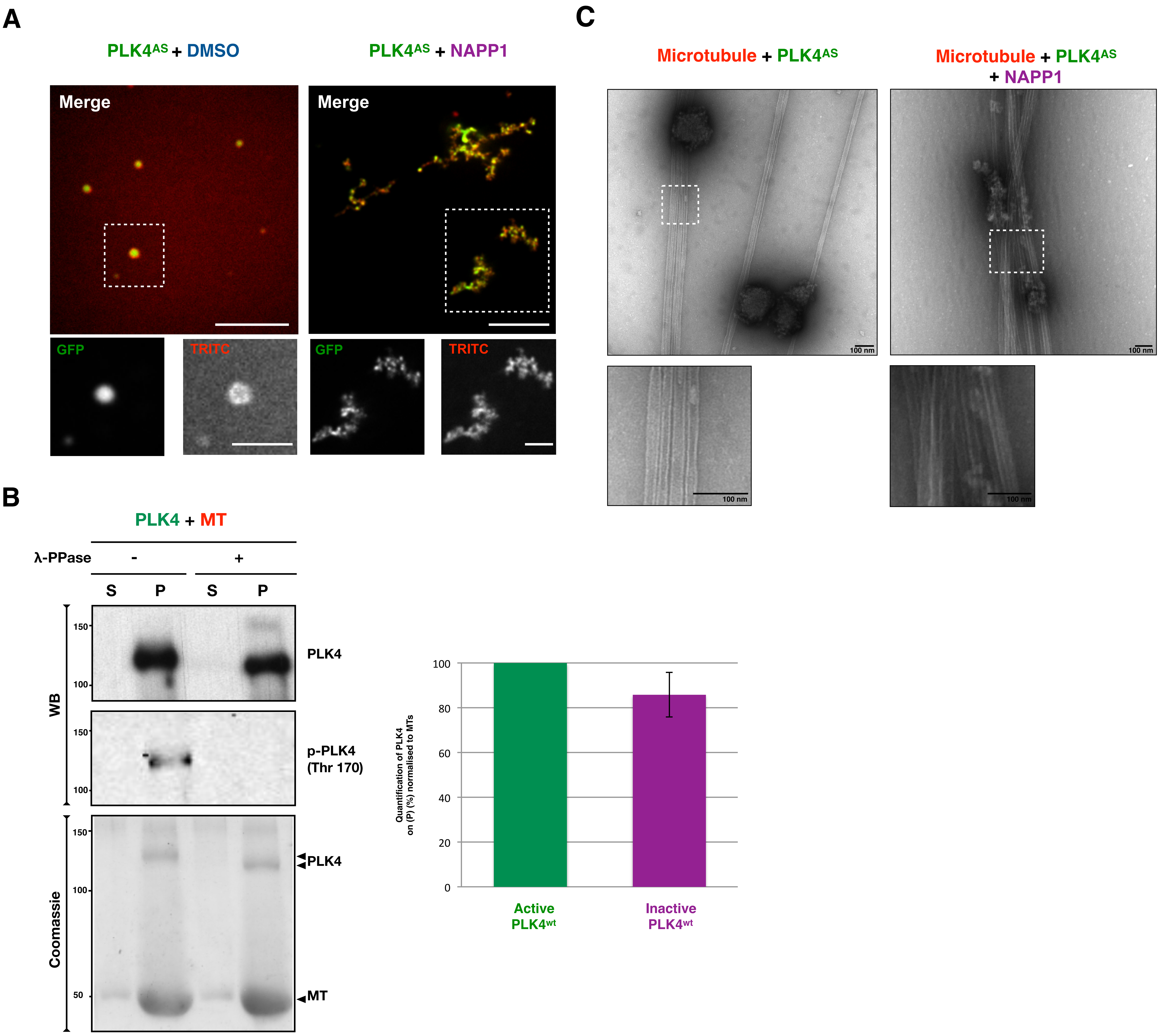

Fig. S2. (Related to Fig. 1 and 2). PLK4 assemblies recruit and bind to MTs independently of PLK4 activity.

(A) Confocal images of PLK4-AS (treated or not with NAPP1) incubated with tubulin-rhodamine TritC. Either active or inactive PLK4 recruits tubulin similarly to the inactivated PLK4 in the presence of MTs. Scale bars: 10 μm; insets: 5 μm. (B) MT pelleting assay. MTs are incubated with PLK4 (active or inactive (treated with λ-PPase)). Coomassie gel and western blots (WB) show supernatant (S) and pellet (P) for each condition. We used anti-PLK4 (for total protein detection) and phospho-Thr-170 (PLK4 activity). A plot of PLK4 quantification o the pellet was performed from three independent experiments. We compared the level of PLK4 treated with λ-PPase to the level of active PLK4 in the pellet. Both PLK4 levels were normalised to MT level in the pellet. (C) EM pictures showing PLK4-AS binding to MTs when treated or not with NAPP1. Scale bars: 100 nm.

Journal of Cell Science • Supplementary information

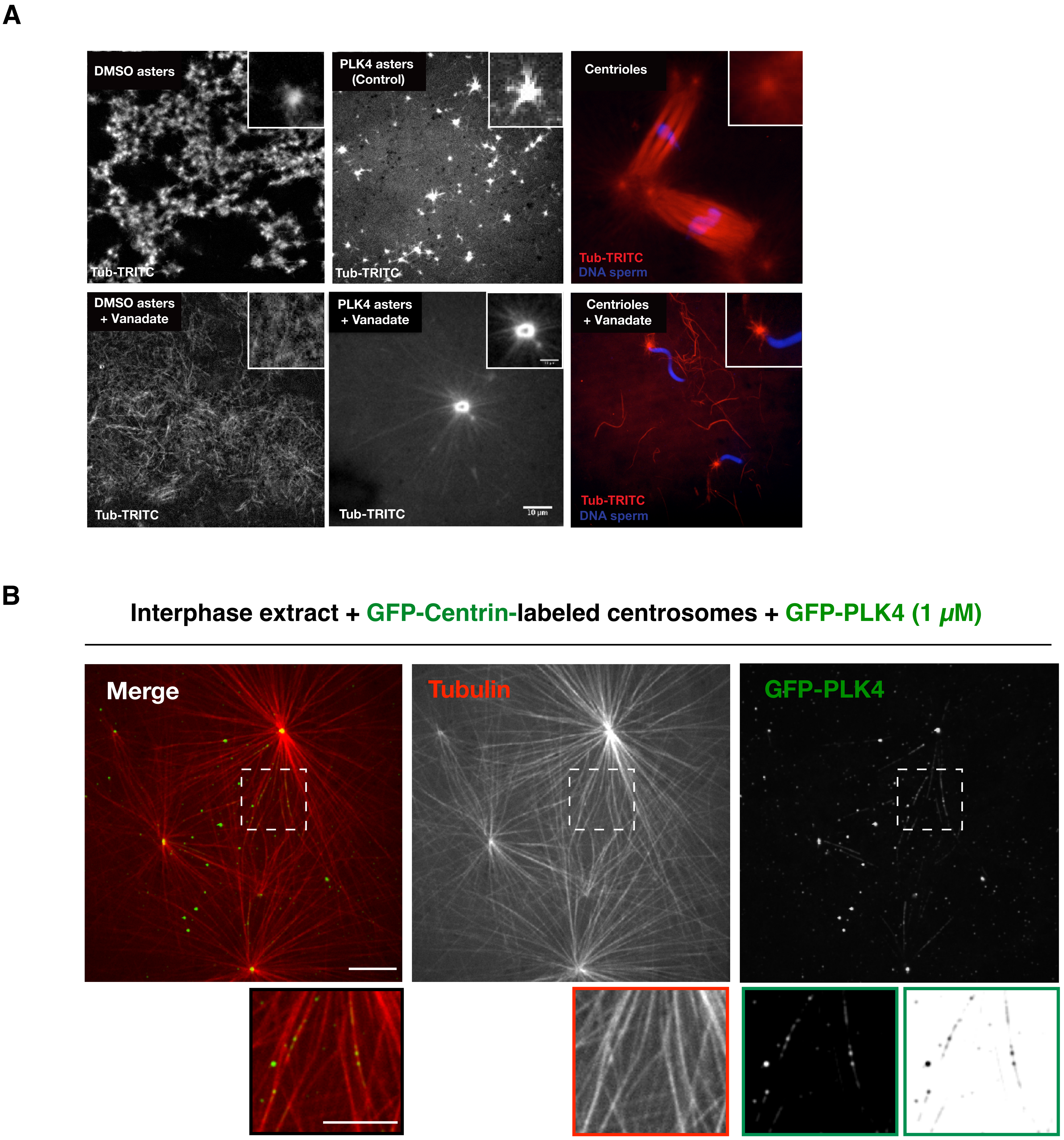

Fig. S3. (Related to Fig. 3). PLK4 binds to Microtubules in *Xenopus* extracts.

(A) Images showing PLK4, DMSO and centriole asters in *Xenopus* extracts in the presence or absence of vanadate. DMSO forms asters in *Xenopus* extracts. Note that those asters are destroyed in the presence of vanadate (dynein inhibitor). Bipolar spindles assemble (red) in *Xenopus* extracts in the presence of sperm, which carries centrioles. In the presence of vanadate bipolar spindles are destroyed, but centrioles still nucleate MTs (positive control). Scale bars: 10 µm. (B) Confocal images showing GFP-PLK4 binding to MTs in interphase extract. Stable asters were obtained by adding GFP-centrin labeled purified centrosome (from HeLa cells) to interphase extracts. Soluble GFP-PLK4 was added to the extract to visualise PLK4 on MTs. Note that even though GFP-PLK4 is mainly at the MTOC centre, PLK4 is also observed on the MTs. Scale bars: 10 µm; insets: 5 µm.

Journal of Cell Science • Supplementary information

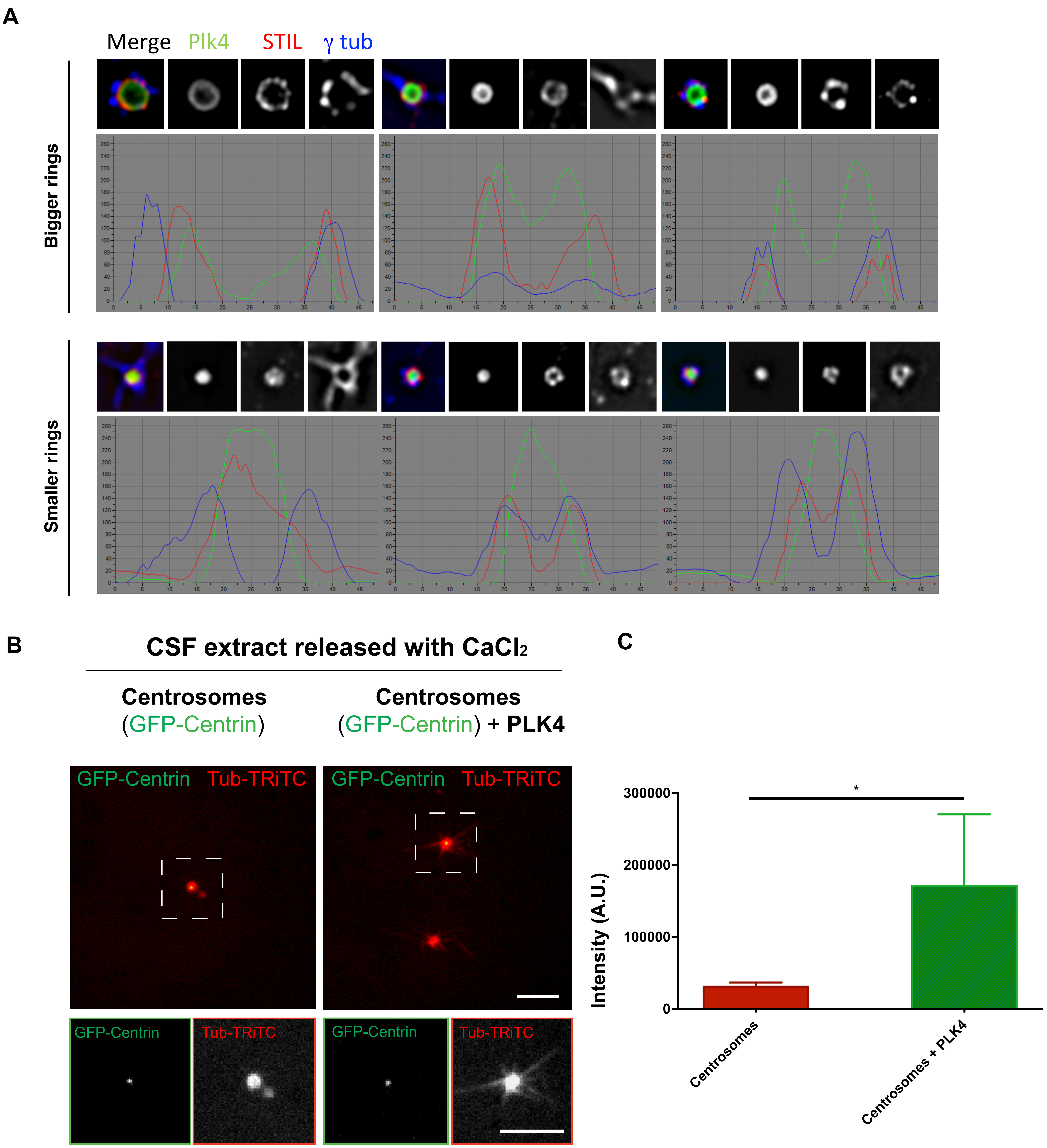

Fig. S4. (Related to Fig 4). PLK4 enhances centrosomal MT nucleation.

(A) Examples of spatial localisation of PLK4, STIL and γ-Tubulin using 3D-SIM of PLK4 assemblies at different sizes (bigger rings and smaller rings). 3D-SIM images of examples of PLK4 assemblies stained with PLK4, STIL and γ-Tubulin. Note that we show examples of PLK4 structures at different sizes and that STIL and γ-Tubulin coat PLK4 assemblies in an organised spatial localisation. (B) Purified centrosomes from HeLa cells labeled with GFP-centrin were added to MII *Xenopus* extracts (CSF) released to interphase with $CaCl_2$. Labeled tubulin was added to visualise asters. Note that the time of incubation is 15 min to visualise only the nucleation that results from centrosomes. GFP-centrin (green) and MTs (red). Scale bars: 5 µm. (C) Quantification of the intensity of centrosomal MT nucleation in MII-extracts released into interphase with $CaCl_2$ in the presence or absence of GFP-PLK4 (1 µM). Results were obtained from four different independent experiments. (A.U: arbitrary units). The statistical data are presented as ± s.d. $^*P < 0.05$, (Student's t-test).

Journal of Cell Science • Supplementary information

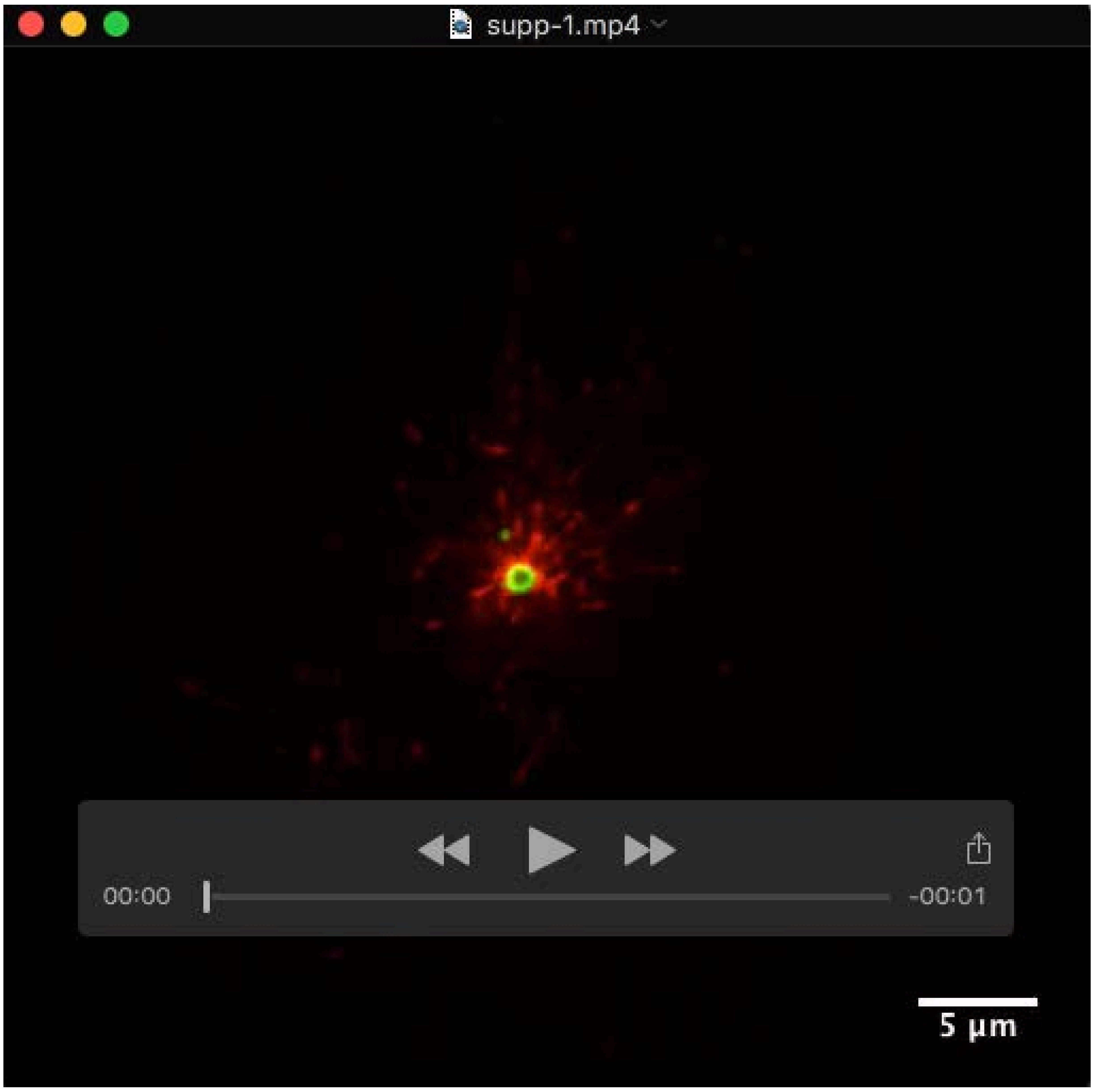

Movie S1 (Related to Fig. 3B). GFP-PLK4 (green) MTOC formation in *Xenopus* egg extract using EB3-mCherry (red) to visualise MT plus ends.

Journal of Cell Science • Supplementary information

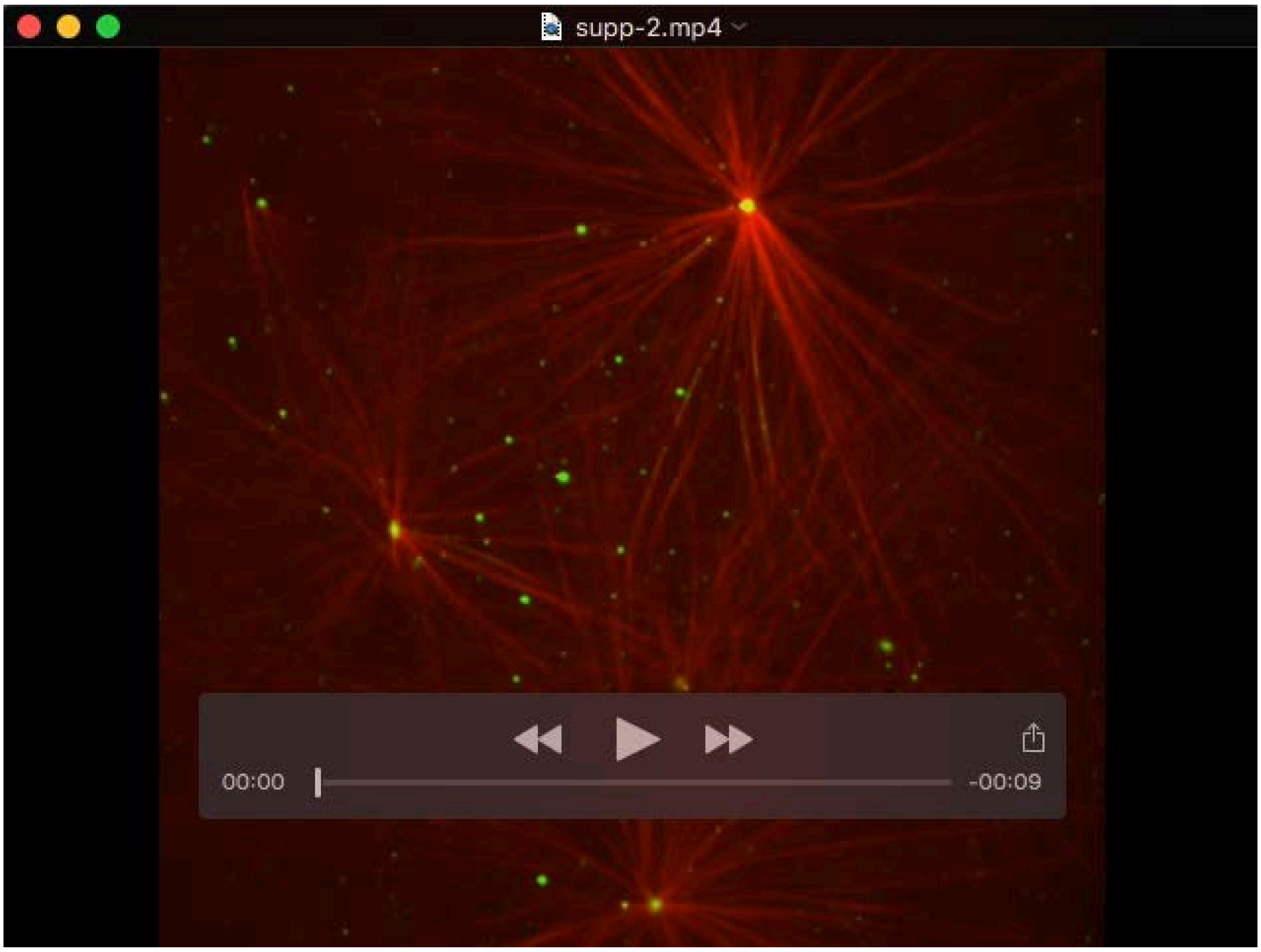

Movie S2 (Related to Fig. 3). GFP-PLK4 (green) binds to MTs (red) in *Xenopus* interphase extracts. We visualise PLK4 at the centre of the aster, and soluble PLK4 was observed sticking to MTs.

Journal of Cell Science • Supplementary information

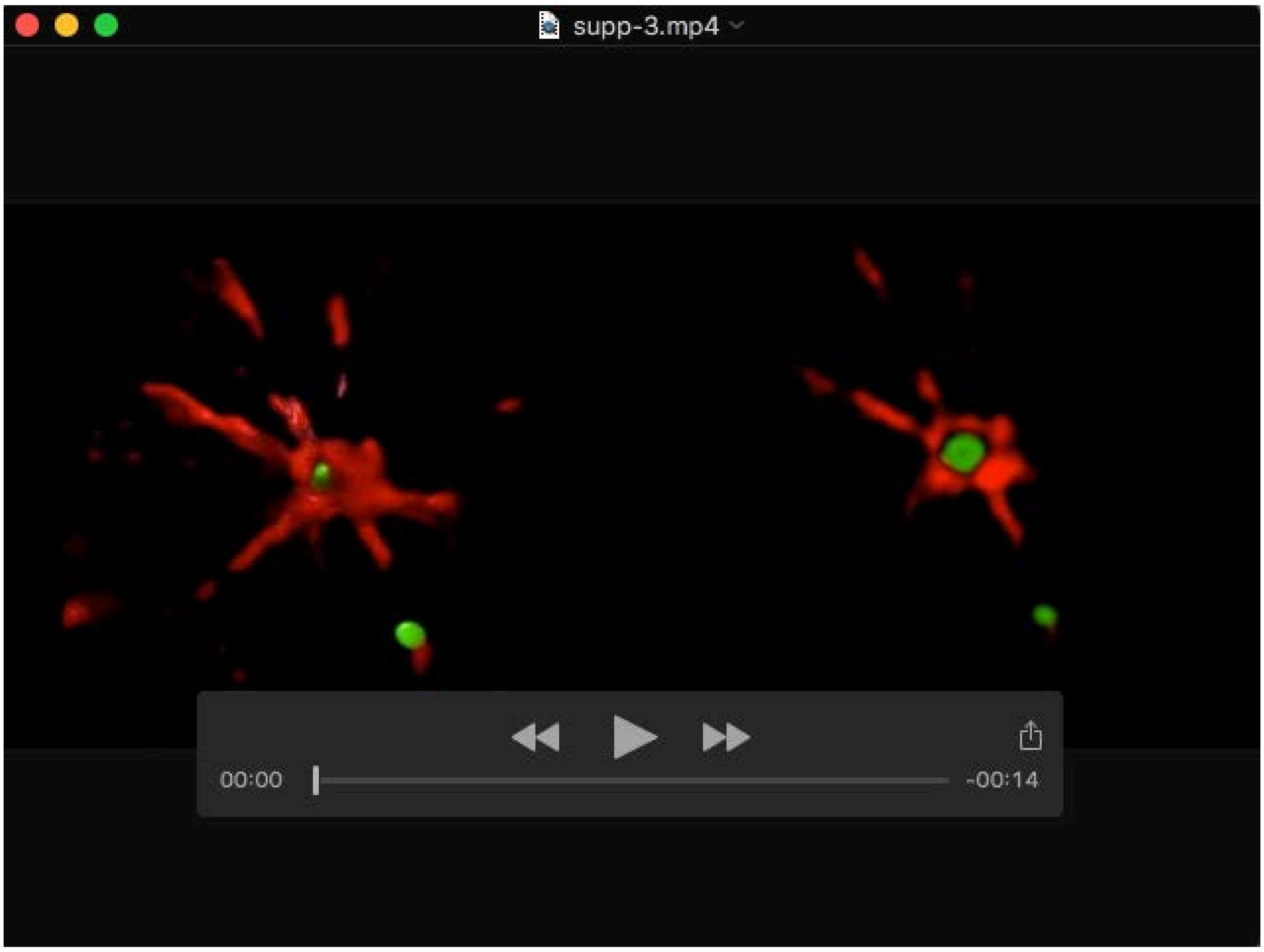

Movie S3 (Related to Fig. 4B). 3D-SIM reconstruction of PLK4 MTOCs in *Xenopus* egg extracts using rhodamine-tubulin. Reconstruction of the whole aster.

Journal of Cell Science • Supplementary information

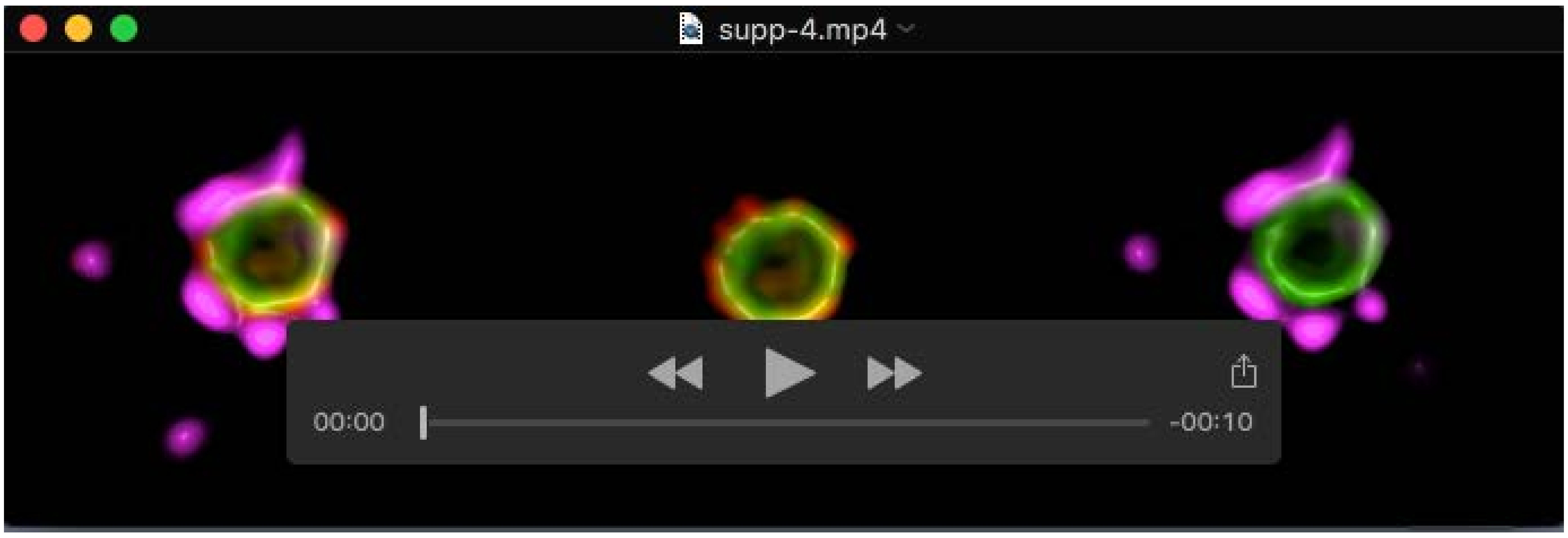

Movie S4 (Related to Fig. 4C) 3D-SIM reconstruction of PLK4 MTOCs in *Xenopus* egg extracts with STIL and γ-tubulin.

Journal of Cell Science • Supplementary information

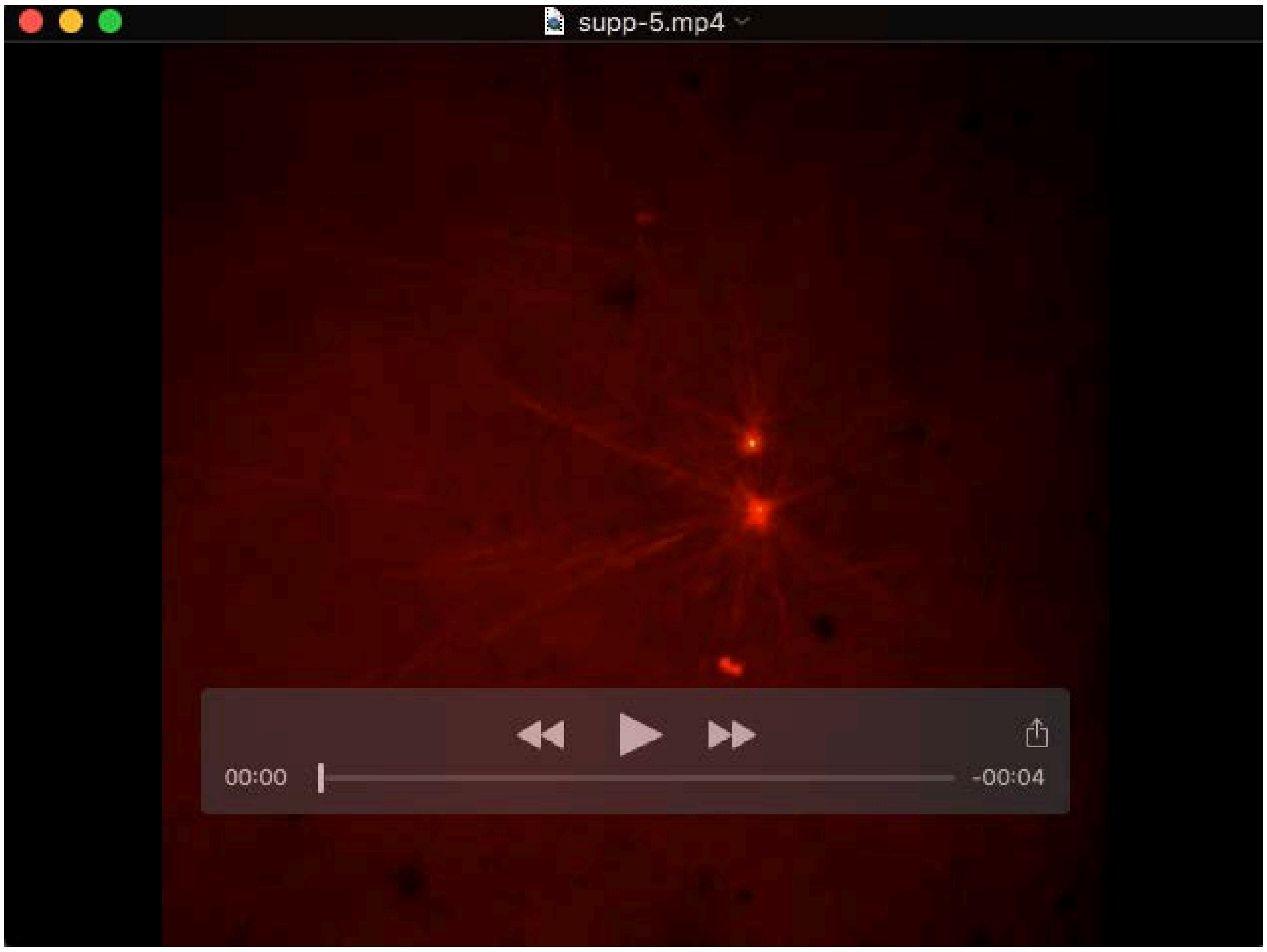

Movie S5 (Related to Fig. 4G). Purified centrioles in *Xenopus* egg extracts.

Journal of Cell Science • Supplementary information

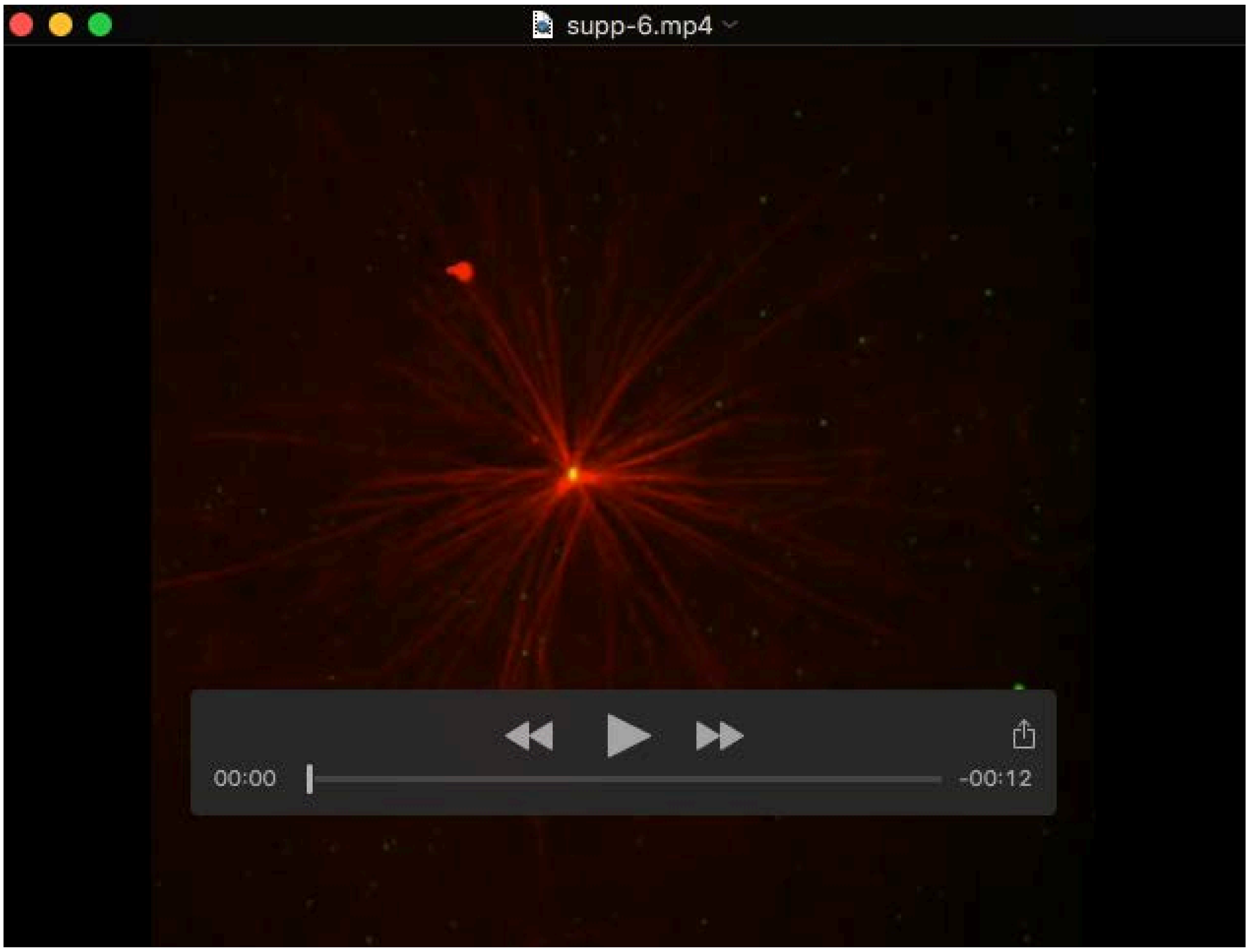

Movie S6 (Related to Fig. 4G). Purified centrioles in *Xenopus* egg extracts in the presence of GFP-PLK4.
